# Why Do HIV Pre-Exposure Prophylaxis Users Discontinue Pre-Exposure Prophylaxis Care? A Mixed Methods Survey in a Pre-Exposure Prophylaxis Clinic in Belgium

**DOI:** 10.1089/apc.2021.0197

**Published:** 2022-04-14

**Authors:** Thibaut Vanbaelen, Anke Rotsaert, Bart K.M. Jacobs, Eric Florence, Chris Kenyon, Bea Vuylsteke, Marie Laga, Reyniers Thijs

**Affiliations:** ^1^ Department of Clinical Sciences and Institute of Tropical Medicine, Antwerp, Belgium.; ^2^ Department of Public Health, Institute of Tropical Medicine, Antwerp, Belgium.; ^3^ Division of Infectious Diseases and HIV Medicine, University of Cape Town, Cape Town, South Africa.

**Keywords:** HIV pre-exposure prophylaxis, PrEP discontinuation, HIV, prevention, PrEP

## Abstract

It remains unclear why patients discontinue HIV pre-exposure prophylaxis (PrEP) care and to what extent they remain at risk for HIV when they do. We reviewed routinely collected medical records and patient questionnaires and performed an e-mail/telephone survey to assess reasons for discontinuing PrEP care, ongoing risks for HIV infection, and associated factors. Patients with more than two registered PrEP visits from a PrEP clinic in Antwerp, Belgium between June 2017 and February 2020 were included in this study. Patients who did not return for a visit after October 30, 2019 and who were not transferred out were considered as having discontinued PrEP care. A total of 143/1073 patients were considered as having discontinued PrEP care. Patients who discontinued PrEP care were more likely to be younger than those who remained in care (35 vs. 38 years old, *p* < 0.01). The most common reasons for discontinuation were having stopped using PrEP (62/101, 61.4%) and “COVID-19” (*n* = 35, 34.7%). The most common reasons for stopping PrEP use was a decreased sexual activity due to coronavirus disease 2019 (COVID-19; 21/62, 33.9%) or not COVID-19 related (10/62, 16.1%), a monogamous relationship (20/62, 32.3%) and consistent condom use (7/62, 11.3%). Among respondents who reported about current HIV risk the majority reported being at low risk either by still taking PrEP (32/91, 35.2%), consistently using condoms, or limiting number of sex acts or partners (58/91, 52.7%). No HIV seroconversion was reported.

## Introduction

HIV pre-exposure prophylaxis (PrEP) is a very effective biomedical intervention to prevent HIV acquisition, when correctly taken.^1–3^ PrEP is recommended for all individuals at substantial risk for HIV infection, such as men who have sex with men (MSM).^1,4,5^ It has been implemented in many countries and its uptake is continuously increasing.^6^ It is estimated that >1,300,000 individuals initiated PrEP in the second quarter of 2021.^7^

PrEP uptake, adherence, and retention in care are needed for PrEP to be effective.^8^ Improving PrEP awareness and uptake are important first steps for effective PrEP implementation and is already extensively studied. Various studies have demonstrated that adherence is crucial for PrEP to be efficacious.^9^ PrEP persistence, the correct and sustained use of PrEP over time, is also critical, but has until now received less scientific attention.^10^

PrEP is not considered a lifelong intervention, but should be taken in periods of increased risk for HIV infection. As HIV risk can vary over time (e.g., due to changes in sexual behavior), PrEP can be discontinued or restarted depending on such risk.^11,12^ On the contrary, discontinuing PrEP during periods of HIV risk is to be avoided given the risk of HIV acquisition.^13–15^

Many PrEP patients discontinuing care do so in the first months after PrEP initiation.^16–18^ One recent review across different countries and populations, including MSM, showed that average retention in PrEP care was 51% after 6 months and 43% after 12 months.^8^ Patients who discontinue PrEP care are more likely to be younger and predominantly choose event-based PrEP.^18,19^ Potential reasons for discontinuing PrEP care are a lower perceived risk for HIV, fear of side effects or experiencing logistical and financial barriers.^17,19^

Recently, the coronavirus disease 2019 (COVID-19) pandemic has been responsible for major changes in sexual behavior and prevention. Sexual risk-taking and PrEP use both declined during the first periods of physical distancing and restriction measures.^20,21^ Hence, the need for PrEP care may have been less in this period. Further, sites where PrEP is provided may have been temporarily closed, or its providers may have been temporarily predominantly occupied in periods requiring more care and attention to COVID-19. Understanding whether or how PrEP care is discontinued in such periods can be important to anticipate subsequent or similar phases of the COVID-19 pandemic.

In Belgium, PrEP is reimbursed through the public health care system since mid-2017 for people at substantial risk for HIV acquisition.^22^ PrEP care is centralized in 12 HIV Reference Centers (HRCs). To get PrEP reimbursed, patients visit an HRC where eligibility is verified (Appendix Table A1). Next, a reimbursement request is submitted to the health insurance fund, which is to be renewed yearly. A total of 4071 persons initiated PrEP before 2020, the vast majority (97.3%) of them being MSM.^23^ Information on PrEP discontinuation, however, is lacking.

The objective of this study was to explore which factors are associated with PrEP care discontinuation and the reasons. An additional objective was to explore to what extent patients who discontinue PrEP care are still at risk for HIV. These insights can complement current knowledge on PrEP care retention and effectiveness of PrEP programs, as well as assess the need for additional tools or interventions to improve retention in PrEP care.

## Methods

### Design

Retrospective analysis of routinely collected medical records and questionnaire data, in addition to a cross-sectional e-mail and telephone survey.

### Sample selection

The setting of this study is a PrEP clinic in Antwerp, Belgium. All patients with more than two PrEP visits between the rollout of PrEP in Belgium (June 01, 2017) and the start of the COVID-19 period (February 28, 2020) were selected. In general, to initiate PrEP, patients first come for a screening visit where information is provided, eligibility for reimbursement is controlled and medically required tests are performed. During a second visit, test results are provided, as well as further counseling and a PrEP prescription.

Data retrieval for this study was November 2020. Patients who did not return for 1 year prior the start of the analysis were theoretically not able to get PrEP reimbursed given the required yearly renewal of the reimbursement. Hence, patients who had not returned since October 30, 2019 were considered as having potentially discontinued PrEP care. Patients who interrupted and re-engaged in PrEP care were not considered as having potentially discontinued PrEP care.

### Data collection

#### Questionnaires

Patients were asked to fill in a questionnaire at each PrEP consultation. For this analysis we used data on sociodemographic characteristics and sexual behavior collected during the first PrEP visit.

#### Medical records

An electronic medical record is held by the health care providers during the PrEP consultation and contains all medically relevant information. The medical records of patients who potentially discontinued PrEP care were examined for a reason for not returning. If during medical records examination, patients were found to have an appointment planned or had a consultation between the censor date and the final analysis, they were not anymore considered as having potentially discontinued PrEP care.

#### Telephone and e-mail survey

Patients who potentially discontinued PrEP care and for whom no reason for not returning was found in the medical records were informed through e-mail about the study and asked consent for an interview. They were provided the option to decline participation or to provide the information directly through e-mail. Patients who did not decline, nor provided information about the reason for not returning, were contacted through telephone for a brief interview about the reasons for not returning for follow-up (FU), PrEP use, HIV testing, and preferred settings for PrEP FU. Respondents could provide multiple answers for each question. The interviews were recorded with patients' consent.

Answers were classified in predefined categories. If no predefined category fitted the answer, it was reported as free text in the category “other.”

Participants were provided the option for a new PrEP appointment at the end of the interview.

### Data analysis

Data concerning the reasons for having discontinued PrEP care, obtained through medical records, e-mail or telephone were grouped into recurring categories and described using absolute numbers and proportions.

Patients who were classified as having potentially discontinued PrEP care (as defined earlier) and who did not report being transferred out in medical records or during the telephone or e-mail survey were considered as having discontinued PrEP care. This category also included patients for whom no information was collected due of lack of contact details or answer. All other patients were considered as having remained in care. We compared patients having discontinued care with those remaining in care to find associations between sociodemographic characteristics, sexual risk factors, and PrEP care continuation.

We defined HIV risk as low if, based on medical records or telephone/e-mail survey, participants reported either still taking PrEP, consistently using condoms, or a limited number of sex acts or partners (being in a monogamous relationship with a HIV-negative or HIV-positive undetectable partner, having no sexual contacts at all, etc.).

Continuous variables were described with mean/median and standard deviation/interquartile range (IQR). Categorical variables were described using proportions. Associations between categorical variables were tested using chi-square and associations between categorical and continuous variables using Student's *t*-test or Mann–Whitney *U* test.

Retention in care was analyzed using survival analysis and Kaplan–Meier curves.

Statistical analysis and graphical representation was performed in R version 4.0.2 (R Foundation for Statistical Computing, Vienna, Austria).

### Ethical approval

The study received ethical approval from the Institutional Review Board of the Institute of Tropical Medicine, Antwerp (IRB 1352–20) and the ethics committee of the University Hospital of Antwerp (18/33/368). Patient consent was asked before filling the questionnaires and before participating in the telephone/e-mail survey. The data were pseudonymized before analysis.

## Results

### Sample selection

**FIG. 1.**
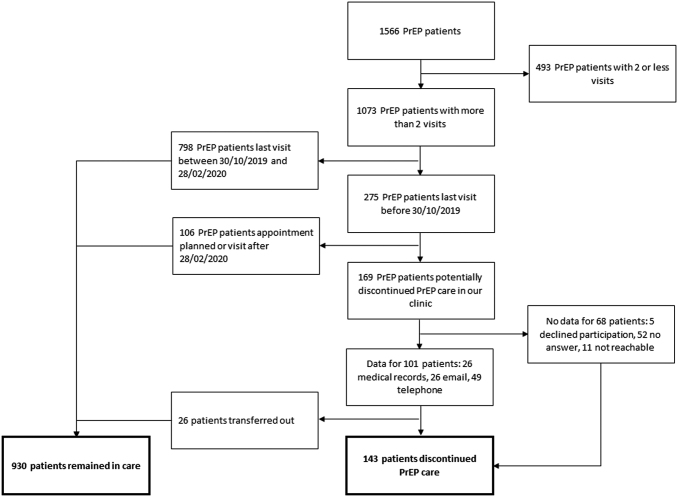
Sample selection.

Among the 1073 selected PrEP patients, 169 were considered as having potentially discontinued PrEP care (Fig. 1). For 26 of those, we found a valid reason for not returning in medical records (e.g., transferred to another clinic, Appendix Table A2) and thus they were not further contacted. We collected data through 49 telephone interviews (Appendix Table A3) and 26 e-mail answers (Appendix Table A4). Eleven patients had missing contact information, 52 patients did not respond, and 5 declined to participate, leading to a response rate of 56.8%. We found that 26 patients were transferred to another center, which brings the final number of patients considered as having discontinued PrEP care to 143 and the number of patients considered having remained in care to 930.

### Retrospective analysis of PrEP questionnaires

The median age of our total sample of patients was 38 years (IQR: 30–46; Table 1) at baseline. The majority was male (99.7%), highly educated (62.2%), of Belgian nationality (85.6%), and from Antwerp province (75.2%). The median number of sex partners in the 3 months preceding the first visit was 6 (IQR: 4–12). Almost all participants (99.3%) had sex with men, 42% reported using party drugs during sex, and 9.8% reported never using condoms for anal sex in the three previous months.

**Table 1.** Sociodemographic Characteristics, Pre-Exposure Prophylaxis Use, and Behavioral Factors

	Total sample^a^	Discontinued PrEP care^b^	Patients remained in care^c^	*p*
	*N* = 1073,* n *(%)	*N* = 143,* n *(%)	*N* = 930,* n *(%)	
Sociodemographics				
Age, years (median; IQR)	38; 30–46	35; 27–44	38; 31–47	**<0.01**
Gender				
Male	1070 (99.7)	143 (100)	927 (99.7)	1
Education^d^				
Higher education	534 (62.2)	62 (55.9)	472 (63.2)	0.39
Country of origin^e^				
Belgium	730 (85.6)	101 (91.8)	629 (84.7)	0.06
Province of origin				
Antwerp	807 (75.2)	99 (69.2)	708 (76.1)	0.09
Sexual practices				
Number of sexual partners previous 3 months (median; IQR)	6; 4–12	6;4–10	7;4–14	0.06
Gender of sexual partners^f^				
Men	952 (99.3)	123 (98.4)	829 (99.4)	0.51
Condom use during anal sex in the previous 3 months^g^				
Never	89 (9.8)	8 (7)	105 (13.2)	0.45
Use of party drugs during sex in the previous 3 months^h^				
Yes	387 (42)	57 (47.5)	330 (41.1)	0.22

Values in bold are significant.

^a^ Total sample of patients having had >2 visits.

^b^ Discontinued PrEP care being defined as having had >2 visits, not returning for FU after 30/10/2019, and not being transferred out.

^c^ Still in care being defined as not belonging to the “discontinued PrEP care” category.

^d^ Missing answers total sample/discontinued PrEP care/patients remained in care: *n* = 215/32/183.

^e^ Missing answers total sample/discontinued PrEP care/patients remained in care: *n* = 220/33/187.

^f^ Missing answers total sample/discontinued PrEP care/patients remained in care: *n* = 114/18/96.

^g^ Missing answers total sample/discontinued PrEP care/patients remained in care: *n* = 165/29/136.

^h^ Missing answers total sample/discontinued PrEP care/patients remained in care: *n* = 151/23/128.

FU, follow-up; IQR, interquartile range; PrEP, pre-exposure prophylaxis.

**FIG. 2.**
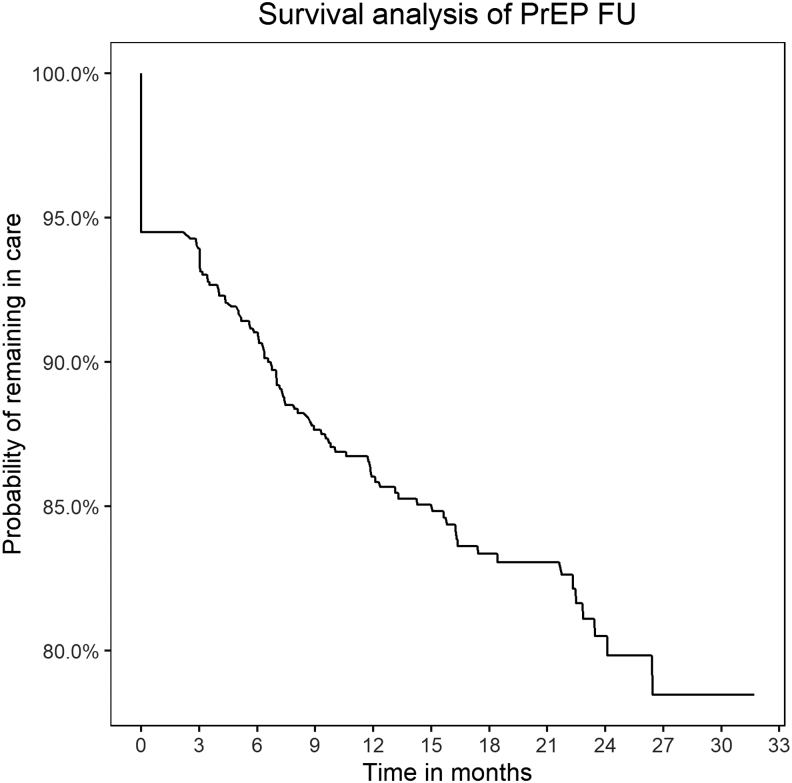
Kaplan–Meier curve of PrEP FU. FU, follow-up; PeEP, pre-exposure prophylaxis.

The survival analysis of PrEP care showed a probability of remaining in FU of 93.9% [95% confidence interval (CI): 92.5–95.4], 91% (95% CI: 89.2–92.9), 87.6% (95% CI: 85.5–89.9), and 86% (95% CI: 83.7–88.4) at 3, 6, 9, and 12 months, respectively (Fig. 2).

Patients who discontinued PrEP care were more likely to be younger when compared with those in care (median age 35 vs. 38, *p* < 0.01, Table 1). There were no significant differences in gender, educational level, country or province of origin, and in sexual practices.

### Results from telephone/e-mail survey and medical records

#### Reasons for PrEP care discontinuation

The most common reasons for discontinuing PrEP care were having stopped using PrEP (*n* = 62, 61.4%, Table 2), COVID-19 (*n* = 35, 34.7%), and being followed up elsewhere (*n* = 26, 25.7%). COVID-19 was responsible for various reasons for PrEP care discontinuation, such as decreased sexual activity, fear to visit the PrEP clinic during the first wave of the pandemic (e.g., avoiding public places), or assuming that health care providers would prioritize COVID-19 over PrEP care, such as the following participant explains:

**Table 2.** Telephone/E-Mail Survey and Medical Records Combined Results

	Total* N* = 101,* n *(%)
Reported HIV protection^a^	
Yes	90 (98.9)
No	1 (1.1)
Reasons for PrEP care discontinuation	
Does not use PrEP anymore	62 (61.4)
COVID-19	35 (34.7)
FU elsewhere	26 (25.7)
Difficulties of access of the clinic (not COVID related)	7 (6.9)
Forgot or missed previous appointment	7 (6.9)
Moved abroad	6 (5.9)
Too many procedures for PrEP FU	4 (3.9)
Side effects	3 (2.9)
No need for FU	2 (1.9)
Death	1 (0.99)

^a^ Defined as still taking PrEP, consistently using condom, or being in a monogamous relationship with a HIV-negative partner or HIV-positive undetectable partner. Denominator = 91, due to lack of information for 10 participants.

COVID-19, coronavirus disease 2019.

“[…] with COVID I thought [the clinic] would have other concerns than PrEP users.” (male, 47 years old, 6 months of FU).

One participant passed away (0.99%) and six had moved abroad (5.9%). Other reasons for PrEP care discontinuation included having forgotten or missed an appointment (*n* = 7, 6.9%), or no longer feeling the need for PrEP FU (*n* = 2, 1.9%). Particular barriers experienced that lead to PrEP care discontinuation included difficulties accessing the clinic (distance, opening hours, etc.; *n* = 7, 6.9%), finding the procedures for PrEP FU too much (*n* = 4, 3.9%), or experiencing side effects of PrEP (*n* = 3, 2.9%) such as the following participant:

“I only used PrEP for a very short time. I took them periodically, usually when I went to the sauna … When I take them, I feel the effect in my body (a kind of rush) .… Usually this feeling is limited, but a few times it was so intense that I had to vomit! So in that respect it was not really a success.” (male, 52 years old, 6 months of FU).

**FIG. 3.**
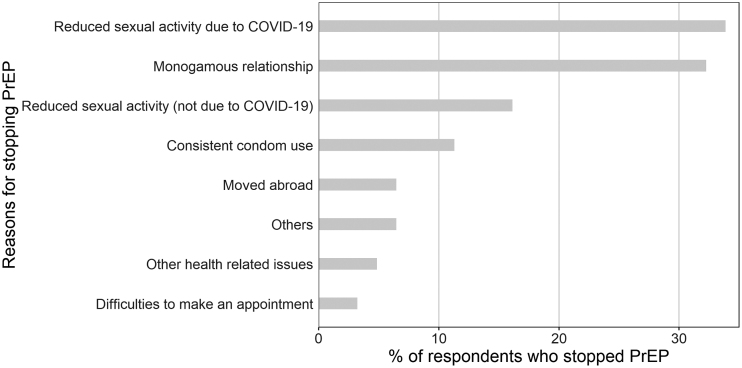
Reasons for stopping PrEP.

Among patients who stopped using PrEP, the majority did so because of a reduced sexual activity due to the COVID-19 pandemic (*n* = 21, 33.9%, Fig. 3) or because of a novel monogamous relationship with a HIV-negative or HIV-positive partner with undetectable viral load (*n* = 20, 32.3%). Other reasons were a reduced sexual activity not related to COVID-19 (*n* = 10, 16.1%), consistent condom use (*n* = 7, 11.3%), having moved abroad (*n* = 4, 6.5%), health-related issues (*n* = 3, 4.8%), and difficulties to make an appointment (*n* = 2, 3.2%). The following participant explained how PrEP well fitted within a particular period of his life:

“I stopped de facto the behavior that made PrEP needed. […] when I was in the 40s [year old, …], for the first time in my life, I started experimenting with drugs to call it that way… and what in lingo is called chemsex […], after 1.5 years or 1 year three quarters, that behavior has almost, but I can in fact say completely, disappeared.” (male, 52 years old, 14 months in FU)

#### Estimated risk for HIV infection when having discontinued PrEP care

No HIV seroconversion was reported among the participants in the e-mail and telephone survey. Among the participants who reported information on HIV risk in the survey or medical records, the vast majority (90/91, 98.9%) reported having a low risk for HIV infection either by still taking PrEP, consistent condom use, or a limited number of sex acts or partners (e.g., being in a monogamous relationship with a HIV-negative or HIV-positive undetectable partner). One participant reported sex acts that were not covered by PrEP nor condoms after his last visit:

“(…) the reason why I went into the PrEP program is because I often travel […] and I was afraid to be contaminated there […] At some point I received an email from [the clinic] and I didn't answer… Then I lost the thread and I think I was unsubscribed from the program, I can't really remember, but it came from my side. (…) I did have risky contacts [after that].” (male, 46 years old, 3.5 months in FU).

## Discussion

We found that the main reason for PrEP care discontinuation was having stopped using PrEP. Among the participants who stopped using PrEP, the majority did so because of a decreased self-perceived risk for HIV. However, particular barriers such as difficulties accessing the clinic or experiencing side effects also lead to patients stopping PrEP use.

The finding that the majority stops using PrEP due to a decreased self-perceived risk for HIV infection is in line with the results of previous studies.^24–28^ However, although these findings sound reassuring, some studies have found that a reduced self-perceived risk for HIV does not always correspond with a real decreased risk for infection.^29,30^ For example, Blumenthal et al. found that 38% of the PrEP patients underestimated their HIV risk and this proportion went up to 90% in people who had a high risk of HIV according to objective criteria.^29^ Interventions focused on improving self-estimation of HIV risk should be explored to allow patients to correctly and safely stop and restart PrEP.

We found that some participants experienced barriers that have led them to discontinue PrEP care, such as (fear of) side effects, difficulties to maintain the PrEP FU schedule, to access the clinic or finding the procedures too much, as found elsewhere.^24–28^ In contrast with other studies,^24–26^ none of the participants reported a financial burden of PrEP as reason for not returning for FU or for stopping with PrEP. This might be due to PrEP being partially reimbursed in Belgium for people at substantial risk for HIV, making it more affordable for people who have access to the public health care system.^22^

Additional interventions or alternative PrEP care delivery models should be explored to address the barriers experienced by PrEP care users and make the thresholds for PrEP care access and persistence as low as possible. For example, PrEP care decentralization or demedicalization as well as new PrEP modalities (e.g., injectable PrEP) are potential interventions to achieve such goals.^31^

Some participants also reported COVID-19 as reason for not discontinuing for PrEP care or stopping PrEP use, either because of a reduced sexual activity imposed by social-distancing measures, or because of fear for public spaces or difficult access of the PrEP clinic. It has been previously described that the early stages of the COVID-19 pandemic have been responsible for major disruptions in PrEP care services as well as changes in sexual behavior and prevention practices.^20,21,32,33^ Further research is needed to assess how these have evolved during the different waves of the pandemic. Interventions aiming at improving retention in care when COVID-19 restrictions are in place, such as telecare, must also be explored.^34^

We found a retention rate of 90.3% at 12 months among the PrEP patients in this clinic. This is much higher than the average 43% reported by a recent review of PrEP care retention, based on studies in various countries and risk groups.^8^ Studies focusing on MSM in the United States also describe a drop in retention rate in the first year after PrEP initiation.^16,17,35^ This discordant finding may be explained by the selection criteria (>2 visits) we applied to define PrEP care discontinuation and because we did not take into account patients who temporarily interrupt PrEP and later re-engaged in care.

Reassuringly, no HIV seroconversion was reported by patients who discontinued PrEP care in our clinic. This contrasts with other studies that found a higher HIV incidence among people who discontinued PrEP.^14,15^ Our finding could be due to the fact that the vast majority of those patients reported being protected against HIV either by still taking PrEP either by a reduced self-perceived risk.

Our study has several limitations. First, our selection criteria include patients who attended more than two PrEP visits, which makes it prone to survival bias and could explain our high retention rate compared with other studies^8^; moreover, due to our sample selection criteria, our total sample is not likely to be representative of all PrEP patients. Second, the e-mail and telephone survey was performed 1–3 years after the last PrEP visit, which could induce recall bias.

Third, the survey was performed by a PrEP care provider of the clinic and answers might have been subject to social desirability bias, as reasons for discontinuing PrEP care directly related to the clinic could have been underreported. Fourth, COVID-19 has been frequently cited as reason for PrEP (care) discontinuation although it does not fit the timeframe of our study. PrEP care discontinuation was defined as not returning after October 30, 2019, when COVID-19 was still out of the picture.

Multiple explanations for this finding are possible. PrEP users do not always attend quarterly visits consistently^18^ and it is not known when exactly the patients stopped PrEP after their last visit. Another explanation could be the fact that multiple answers for the same question could be provided by the patients, whereas COVID-19 could be a reason for not having restarted PrEP at the time of the survey it might not have been the reason for stopping PrEP initially.

Moreover, due to insufficient detail about the reasons for discontinuation it was not always clear what the main reason was, for example, having limited access due to self-quarantine, or limited availability of services. Also, sometimes the discontinuation was multi-factorial, making it impossible to distinguish between reasons, for example, no more need for PrEP due to reduced sexual contacts, or reduced sexual contacts due to COVID-19 restrictions. Fifth, we did not perform HIV testing and HIV seroconversion data are based on self-reporting during the telephone/e-mail survey. Finally, we could not obtain information from all patients who discontinued PrEP care in our clinic, which makes our sample not likely to be representative of all patients who discontinued PrEP care.

These limitations notwithstanding, this study showed that, although PrEP patients in our study discontinued PrEP care for various reasons, most of them thought to be at low risk for HIV infection when doing so. It is known that PrEP can be discontinued during periods at lower risk for HIV and restarted should the risks reappear.^11^ It is crucial that patients correctly estimate their risk for HIV infection to safely decide when and how to take PrEP. Alternative or novel strategies are also required to address potential barriers to PrEP care, particularly in times of COVID-19 where sexual activities and prevention services face many disruptions.

## Appendix

**Appendix Table A1:** Eligibility Criteria for Reimbursement of Pre-Exposure Prophylaxis in Belgium^22^

Criteria for the reimbursement of PrEP in Belgium are as follows:
MSM at very high risk of HIV infection:
People who have had unprotected anal sex with at least two partners in the past 6 months
People who have had multiple STDs (syphilis, chlamydia, gonorrhea, or primary hepatitis B or C infection) in the past year
People who have used PEP more than once a year
People who use psychoactive substances during sexual activity
High-risk individuals with individual risk:
PWID (people who inject drugs) who share needles
People in prostitution who are exposed to unprotected sex
People in general exposed to unprotected sex at high risk of HIV infection
Partner of an HIV-positive patient without viral suppression (newly on treatment or no viral suppression with adequate treatment)

MSM, who have sex with men.

**Appendix Table A2.** Reasons for Pre-Exposure Prophylaxis Care Discontinuation Retrieved from Medical Records

	Medical records* N* = 26,* n *(%)
Side effects	1 (3.9)
COVID-19 (did not wish to come to the clinic)	1 (3.9)
Death	1 (3.9)
Transfer HIV clinic	7 (26.9)
Stopped taking PrEP	14 (53.9)
Moved abroad	2 (7.7)

**Appendix Table A3.** Results LTFU Telephone Interviews

	Telephone interviews,* n *(%)
HIV risk (*N* = 49)	
Seroconversion	
Yes	0 (0)
No	49 (100)
Protection^a^	
Yes	48 (97.9)
No	1 (2)
Reasons for PrEP care discontinuation (*N* = 49)	
Stopped using PrEP	32 (65.3)
FU elsewhere	13 (26.5)
No need for FU	1 (2)
Forgot or missed previous appointment	5 (10.2)
Difficulties of access of the clinic (not COVID related)	6 (12.2)
Too many procedures for PrEP FU	2 (4)
COVID-19	22 (44.9)
Death	1 (2)
Moved abroad	4 (8.1)
Reasons for stopping PrEP (*N* = 32)	
Monogamous relationship	8 (25)
Reduced sexual activity due to COVID-19	17 (53.1)
Consistent condom use	4 (12.5)
Others	4 (12.5)
Reduced sexual activity (not due to COVID-19)	6 (18.8)
Difficulties to make an appointment	2 (6.3)
Moved	3 (9.4)
Other health-related issues	3 (9.4)
FU of those still taking PrEP (*N* = 17)	
Followed up in another PrEP clinic	11 (64.7)
Followed up by GP	2 (11.8)
Still had PrEP pills	4 (23.5)
COVID-19 reasons for discontinuing PrEP care (*N* = 22)	
Reduced sexual contacts	19 (86.4)
Did not wish to come to the clinic	3 (13.6)
Blocked abroad	1 (4.5)
Difficulty to make an appointment	1 (4.5)
New appointment given	
Yes	11 (22.4)
No	38 (77.6)

^a^ Defined as either reporting still taking PrEP, having no risks or systematically using condoms.

COVID-19, coronavirus disease 2019; FU, follow-up; LTFU, lost to follow-up; PrEP, pre-exposure prophylaxis.

**Appendix Table A4.** Results LTFU E-Mail Answers

	E-mail answers,* n *(%)
Protection^a^ (*N* = 22)	
Yes	20 (91)
No	0 (0)
Not specified	2 (9)
Reasons for PrEP care discontinuation (*N* = 26)	
COVID-19	12 (46.1)
Does not use PrEP anymore	16 (61.5)
FU elsewhere	6 (23.1)
Missed or forgot previous appointment	2 (7.7)
Difficulties of access of the clinic (not COVID-19 related)	1 (3.9)
No need for FU	1 (3.9)
Side effects	1 (3.9)
Too many procedures for PrEP FU	2 (7.7)
New appointment given	
Yes	3 (11.5)
No	23 (88.4)

^a^ Defined as either reporting still taking PrEP, having no risks, or systematically using condoms.

LTFU, lost to follow-up.
